# Fatores Associados à Recorrência na Síndrome de Takotsubo: Uma Revisão Sistemática

**DOI:** 10.36660/abc.20180377

**Published:** 2020-04-06

**Authors:** Felipe Alverenga Duarte Campos, Luiz Eduardo Fonteles Ritt, João Paulo Soares Costa, Constança Margarida Cruz, Gilson Soares Feitosa-Filho, Queila Borges de Oliveira, Eduardo Sahade Darzé

**Affiliations:** 1 Escola Bahiana de Medicina e Saúde Pública SalvadorBA Brasil Escola Bahiana de Medicina e Saúde Pública, Salvador, BA – Brasil; 2 Hospital Cárdio Pulmonar SalvadorBA Brasil Hospital Cárdio Pulmonar, Salvador, BA – Brasil; 3 Hospital Santo Antônio Obras Sociais Irmã Dulce SalvadorBA Brasil Hospital Santo Antônio - Obras Sociais Irmã Dulce, Salvador, BA – Brasil; 4 Faculdade de Tecnologia e Ciências Curso de Medicina SalvadorBA Brasil Faculdade de Tecnologia e Ciências, Curso de Medicina, Salvador, BA – Brasil

**Keywords:** Síndrome de Takotsubo, Cardiomiopatia de Takotsubo, Recidiva

## Abstract

**Fundamento:**

A síndrome de Takotsubo (STT) é caracterizada por uma disfunção sistólica temporária do ventrículo esquerdo (VE) relacionada a um evento estressante. No entanto, os fatores associados à sua recorrência ainda não estão bem estabelecidos.

**Objetivo:**

Analisar os principais fatores associados à recorrência da STT.

**Métodos:**

Uma revisão sistemática foi realizada usando o modelo PRISMA. Foram incluídos estudos observacionais, publicados entre janeiro de 2008 e outubro de 2017, que apresentaram uma taxa de recorrência de pelo menos 3% e/ou 5 ou mais pacientes com recidiva e que preencheram pelo menos 80% dos critérios STROBE.

**Resultados:**

Seis artigos atenderam aos critérios para esta revisão sistemática. A taxa de recorrência variou de 1 a 3,5% ao ano (taxa de recorrência global 3,8%). Um estudo associou maior taxa de recorrência ao sexo feminino, quatro relataram o tempo entre o primeiro e o segundo episódio, um estudo associou o índice de massa corporal (IMC) e a hipercontratilidade da parede anterior média do VE a uma maior taxa de recorrência. Não foi determinada associação entre recorrência e alterações eletrocardiográficas. O uso de betabloqueadores não foi associado a taxas de recorrência.

**Conclusões:**

Sexo feminino, tempo desde o primeiro episódio da síndrome, baixo IMC e obstrução ventricular foram relatados como possíveis preditores de recorrência da STT.

## Preâmbulo e relato de caso

Uma paciente do sexo feminino, com 62 anos de idade, foi admitida para cirurgia eletiva de ritidoplastia e blefaroplastia. Ela pesava 61 kg e tinha 1,65 m de altura, com história de glaucoma e hipotireoidismo. Ela foi considerada de baixo risco cardiovascular para o procedimento, sem histórico pessoal ou familiar de doença cardiovascular. Durante a cirurgia, sob anestesia geral, a paciente apresentou ritmo idioventricular, seguido de choque circulatório e parada cardiorrespiratória. Ela foi ressuscitada com sucesso e seu eletrocardiograma (ECG) mostrou um padrão de elevação de ST nas derivações laterais. Foi prontamente submetida a um cateterismo cardíaco que não apresentou lesão coronariana, mas acinesia nas paredes ventriculares apicais e mediais e hipercinesia das partes basais, padrão semelhante à Síndrome de Takotsubo (STT). Esse padrão foi confirmado em um eletrocardiograma, que mostrou uma fração de ejeção de 40%. Após ajustes na terapia da insuficiência cardíaca, ela recebeu alta hospitalar em 10 dias, clinicamente estável. Em seis meses, ela já havia recuperado suas funções globais e segmentares. Após 1 ano, ela planejava se submeter a outra cirurgia plástica e perguntou sobre o risco de recorrência. Essa questão foi a principal motivação desta revisão sistemática.

## Introdução

A STT, também denominada cardiomiopatia de Takotsubo ou síndrome do coração partido,^1^^,^^2^ é caracterizada por disfunção sistólica e diastólica temporária do ventrículo esquerdo, geralmente associada a um evento de grande estresse emocional ou físico. Apresenta-se clinicamente com dor torácica aguda, dispneia, alterações eletrocardiográficas e presença de biomarcadores de lesão cardíaca elevada, sendo muito semelhante a uma síndrome coronariana aguda, apesar da ausência de estenose coronariana significativa relacionada à área afetada.^3^ Estima-se que cerca de 2% dos pacientes com suspeita de síndrome coronariana aguda tenham TTS.^4^

As mulheres na pós-menopausa são o grupo mais afetado por essa condição, provavelmente devido a problemas hormonais, embora homens e jovens também possam ter TTS. Sugere-se que a fisiopatologia da doença esteja relacionada a uma descarga grande e abrupta de catecolaminas.2 Portanto, o uso de betabloqueadores (BB) tem sido proposto como estratégia de prevenção.^3^

O prognóstico é geralmente bom e caracterizado como benigno por muitos autores, embora exista um risco de 1-2% de arritmias ventriculares e aproximadamente 2% de mortalidade intra-hospitalar associada à STT.^5^ Pacientes com histórico de STT têm taxa de recorrência anual de 1,5%, embora possa chegar a 11% em 4 anos.^3^^,^^5^^,^^6^

Nos últimos anos, houve um aumento no número de estudos relacionados à TTS publicados, especialmente nos EUA, Europa e Japão. Muitos dados sobre essa patologia provêm do Registro Internacional Takotsubo (Registro InterTAK), uma rede internacional de colaboração com dados de 35 centros cardiovasculares em 15 países diferentes.^2^^,^^7^ No entanto, os preditores de recorrência da STT ainda não estão bem estabelecidos.

## Objetivo

O presente estudo teve como objetivo analisar os principais fatores associados à recorrência da STT.

## Métodos

Foi proposta uma revisão sistemática da literatura, utilizando o modelo PRISMA. Foram pesquisadas as principais bases de dados da literatura internacional – PubMed, Scielo, Lilacs e Cochrane.

Como estratégia de busca, foram utilizados os seguintes descritores: Síndrome de Takotsubo; Síndrome do Balão Apical do Ventrículo Esquerdo; Cardiomiopatia de Takotsubo; Cardiomiopatia por estresse; Síndrome do coração partido. Utilizou-se a ferramenta PubMed MeSH, adicionando Recorrência como descritor complementar.

Estratégia para seleção de artigos: a seleção foi realizada em outubro de 2017. Todos os artigos publicados entre janeiro de 2008 e outubro de 2017 foram incluídos inicialmente para posterior apreciação. Primeiramente, os títulos foram avaliados, seguidos do resumo e, finalmente, foi realizada uma análise cuidadosa do artigo completo, a fim de identificar sua qualidade e relevância para o objetivo proposto. Foram utilizados os critérios de Reforço dos Relatórios de Estudos Observacionais em Epidemiologia (STROBE)^8^ para avaliar a qualidade metodológica dos estudos observacionais, com um mínimo de 80% de conformidade com os 22 itens da lista de verificação do STROBE a serem incluídos neste estudo. Todo esse processo foi realizado por dois pesquisadores. Os artigos também foram pesquisados com base nas referências dos artigos selecionados.

Foram selecionados apenas artigos caracterizados como estudo de coorte prospectivo, estudo de coorte retrospectivo, controle de caso ou estudo de série de casos. Artigos em que o texto não estava em inglês foram excluídos. Apenas estudos relatando pelo menos 3% de taxa de recorrência e/ou cinco ou mais pacientes com recorrência foram incluídos, para que pudesse haver uma análise significativa dos preditores de recorrência.

## Resultados

**Figura 1:**
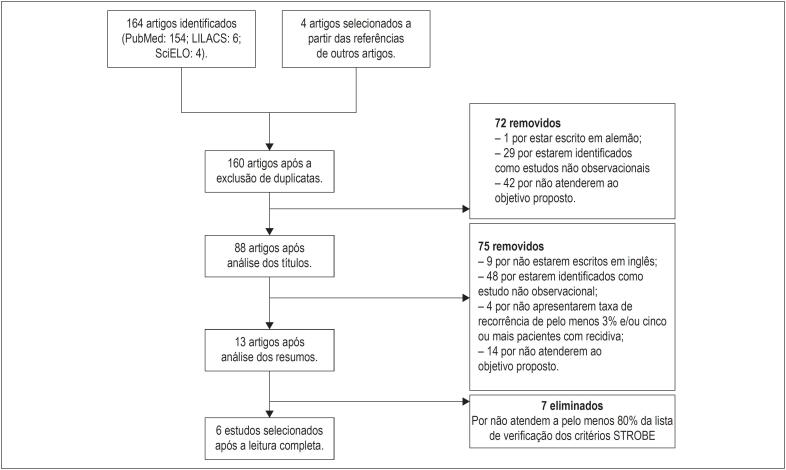
Fluxograma Prisma da seleção dos estudos para a composição da revisão sistemática.

Inicialmente, foram identificados 164 artigos. Quatro outros estudos foram identificados e selecionados a partir das referências dos artigos inicialmente identificados. Ao final da análise dos estudos, seis foram selecionados para compor essa revisão sistemática (Figura 1 e Tabela 1).

**Tabela 1:** Escore e porcentagem da qualidade dos artigos com base nos critérios do STROBE (*Strengthening the Reporting of Observational Studies in Epidemiology*)

Referência	Design do estudo	Publicação	Pontos no STROBE	%
Looi et al.,^9^	Coorte prospectiva.	Journal of Heart, Lung and Circulation.	18	81,8
Templin et al.,^3^	Caso-controle.	The New England Journal of Medicine.	19	86,3
Patel et al.,^10^	Coorte retrospectiva.	Journal of Cardiac Failure.	19	86,3
Elesber et al.,^11^	Coorte retrospectiva.	Journal of the American College of Cardiology.	19	86,3
Vriz et al.,^12^	Coorte prospectiva.	Journal of Cardiovascular Medicine.	18	81,8
Nishida et al.,^13^	Caso-controle.	Heart and Vessels.	20	90,9

Globalmente, a taxa de recorrência, antes de excluir os quatro artigos que não atendiam aos critérios de pelo menos 3% de taxa de recorrência e/ou cinco ou mais pacientes com recorrência, variou de 0,2 a 5% ao ano. A taxa de recorrência global, considerando os estudos selecionados, foi de 3,8% em um seguimento que variou de 5 a 17 anos.

A tabela 2 traz as informações compiladas dos estudos selecionados. Looi et al.^9^ estudaram um estudo de coorte prospectivo de 100 pacientes diagnosticados com STT pelos critérios de Mayo. Desses, sete pacientes (7%) apresentaram recidiva e um apresentou quatro episódios recorrentes. As recorrências ocorreram entre 99 e 679 dias após o primeiro episódio. Todas as recidivas ocorreram dentro de dois anos após o primeiro episódio, sendo mais frequentes no primeiro ano. Em quatro dos sete pacientes que apresentaram recorrências (57%), os eventos iniciais e subsequentes foram desencadeados por estresse emocional. Quatro dos sete pacientes que tiveram recorrências já estavam usando um BB no segundo episódio.

**Tabela 2:** Características dos estudos selecionados

Referência	População estudada	Nível de significância adotado	Taxa de recorrência (recorrência N/N total)	Dados analisados com possível associação de recorrência
Looi et al.,^9^	Pacientes internados no Middlemore Hospital, Auckland City Hospital e North Shore Hospital, Auckland, Nova Zelândia.	p < 0,05	7% (7/100)	– Tempo entre manifestações: as recidivas ocorreram de 99 a 679 dias após o primeiro episódio, sendo mais frequentes no primeiro ano. – Fator desencadeante: 57% dos pacientes com recidiva apresentaram estresse emocional como gatilho. - Características clínicas: a recorrência nos pacientes com supradesnivelamento do segmento ST não foi maior quando comparada aos pacientes que não apresentaram supradesnivelamento do segmento ST (7,4 e 6,3%, respectivamente); p = 1,00. - Medicações em uso: 4 (57%) dos pacientes usaram BB na recorrência.
Templin et al.,^3^	Pacientes obtidos através do banco de dados da Clínica Mayo. Pacientes com SCA de Zurique Registro de Síndrome Coronariana Aguda.	p < 0,05	3,26% (57/1750)	- Tempo entre manifestações: a recorrência ocorreu de 25 dias a 9,2 anos após o primeiro episódio. - Medicações em uso: 29 pacientes (50,8%) usaram BB durante o segundo episódio.
Patel et al.,^10^	Pacientes obtidos através do banco de dados da Clínica Mayo.	p < 0,05 para homens *versus* mulheres p < 0,25 para comparação com mulheres ≥ 50 anos de idade (devido a comparações múltiplas)	3,13% (7/224)	– Sexo: não houve recidivas em homens e todas as 7 recidivas foram em mulheres (14,8%) - Idade: a recorrência em mulheres com idade < 50 anos foi mais prevalente em relação à recorrência em mulheres com idade ≥ 50 anos (16 e 3%, respectivamente; p = 0,017).
Elesber et al.,^11^	Pacientes diagnosticados com STT submetidos ao banco de dados do centro de cateterismo da Clínica Mayo.	p < 0,05	11,4% (10/100)	– Tempo entre manifestações: 4,4 ± 4,6 anos em média entre os episódios, com maior taxa de recorrência nos primeiros 4 anos em comparação aos anos subsequentes (2,9 e 1,3% ao ano, respectivamente). - Medicações em uso: recorrência em pacientes em uso X sem uso de: aspirina (60x67%), p = 0,67; IECA/BRA (60x51%), p = 0,59; BB (80x52%), p = 0,10; Estatinas (40x33%), p = 0,67.
Vriz et al.,^12^	Pacientes no Hospital Comunitário de San Antonio (San Daniele del Friuli, Udine, Itália).	p < 0,05	21,7% (5/23)	- Idade: mais frequente em pacientes idosos. – Tempo entre manifestações: a recorrência ocorreu em média 105,4 ± 82,92 dias após o primeiro episódio, sendo mais frequente nos primeiros 3 meses. - Características clínicas: recorrência mais frequente em pacientes com menor FEVE, PAS mais baixa e pico de troponina mais alto. - Medicações em uso: a terapia com BB não impediu a recorrência.
Nishida et al.,^13^	Pacientes do banco de dados do Registro BOREAS.	p < 0,05	2,8% (7/251)	Características clínicas: IMC baixo, hipercontratilidade ventricular média e comprometimento ventricular direito foram associados a uma maior taxa de recorrência da STT (p = 0,048, 0,01 e 0,06, respectivamente). As HRs de recorrência para IMC (por aumento de 1 kg/cm2) e OMV foram 0,75 (IC 95% 0,54–0,99) e 14,71 (IC 95% 1,87–304,66), respectivamente.

BB: betabloqueador; SCA: síndrome coronariana aguda; TTS: síndrome de Takotsubo; IECA: inibidor da enzima de conversão da angiotensina; BRA: bloqueador do receptor da angiotensina II; FEVE: fração de ejeção do ventrículo esquerdo; PAS: pressão arterial sistólica; IMC: índice de massa corporal; HR: taxas de risco; IC: intervalo de confiança; OMV: obstrução microvascular.

Templin et al.^3^ apresentaram um estudo caso-controle com 1.750 pacientes com STT, segundo os critérios de Mayo. Desses, 455 pacientes foram pareados por idade e sexo com pacientes diagnosticados com síndrome coronariana aguda (SCA) e que tiveram seus dados obtidos no Registro de Síndrome Coronariana Aguda de Zurique. Durante um período de acompanhamento de 17 anos, 57 pacientes com STT tiveram recorrências, representando uma taxa de recorrência de 1,8% por paciente-ano. O segundo episódio ocorreu de 25 dias a 9,2 anos após o primeiro. Um total de 29 dos 57 pacientes com recorrências (50,8%) estavam em terapia com BB no momento do episódio recorrente.

No estudo de coorte retrospectivo de Patel et al.^10^ 224 pacientes diagnosticados com STT tiveram seus dados obtidos no banco de dados da Clínica Mayo por um período de 10 anos. Apenas 7 episódios recorrentes foram documentados. Nenhum dos homens teve recorrência do STT. Durante um seguimento médio de 3,5 anos, 2 mulheres com menos de 50 anos (16%) e 5 mulheres com 50 anos ou mais (3%) desenvolveram recorrência do STT (p = 0,017).

Elesber et al.^11^ estudaram uma coorte retrospectiva e analisaram dados de 100 pacientes diagnosticados com STT ao longo de um período de 16 anos e 11 meses. A taxa de recorrência foi de 11,4% em um tempo médio de acompanhamento de 4,4 ± 4,6 anos, sendo maior nos primeiros 4 anos (2,9% ao ano) e diminuindo para cerca de 1,3% ao ano no tempo subsequente do segmento. Não houve diferença entre os pacientes com ou sem recidiva em relação ao uso de: aspirina (60 *versus* 67%; p = 0,67); inibidor da enzima de conversão da angiotensina (IECA)/bloqueador do receptor da angiotensina II (BRA) (60 *versus* 51%; p = 0,59); BB (80 *versus* 52%; p = 0,10); ou estatinas (40 *versus* 33%; p = 0,67).

Em outro estudo prospectivo de coorte,^12^ 23 pacientes submetidos a angiografia coronariana foram diagnosticados com STT de acordo com os critérios de Mayo, por um período de 7 anos e 11 meses. Cinco pacientes (21,7%) desenvolveram STT recorrente e um paciente apresentou 2 episódios recorrentes. O tempo médio para um episódio recorrente foi de 105,4 ± 83 dias, e a taxa de recorrência foi maior nos três primeiros meses. Comparados com pacientes sem recorrência, aqueles com episódio recorrente eram mais velhos (71,4 *versus* 65,7 anos), apresentavam menor fração de ejeção (36,5 *versus* 44,2%), pressão arterial sistólica mais alta (139 *versus* 128,4 mmHg) e níveis mais altos de troponina (8,1 *versus* 2,5 μg/ml). Três dos cinco pacientes que apresentaram recorrência estavam sob uso de BB.

Nishida et al.^13^ apresentaram um estudo caso-controle. Foram analisados dados de 251 pacientes que compuseram o Registro BOREAS (com 15 países participantes) de junho de 1999 a março de 2012. Os pacientes foram divididos em dois grupos, aqueles com balão apical (tipo A), apresentação clássica de STT e aqueles com balão não apical (tipo não A), que incluíram todas as outras formas de apresentação da síndrome. Durante um seguimento de 2,6 ± 2,8 anos, a taxa de recorrência foi de 2,8% (7/251), sem diferença significativa entre os grupos A e não-A (2,8 e 2,9%, respectivamente). Na análise univariada, o baixo Índice de Massa Corporal (IMC) (p = 0,048), ventricular médio (p = 0,01) e o comprometimento concomitante do ventrículo direito (p = 0,06) foram associados à recorrência do STT. Somente o IMC (razão de risco [HR] 0,75; intervalo de confiança [IC] de 95% 0,54–0,99; p = 0,048) e obstrução do meio do ventrículo (HR 14,71; IC 95% 1,87–304,66; p = 0,01) permaneceram significativamente associados à recorrência do STT.

## Discussão

A taxa de recorrência da STT é variável na literatura e os fatores associados a ela também não foram claramente definidos. Essa afirmação ficou clara quando, recentemente, aquela paciente de 62 anos de idade, com histórico de ressuscitação para parada cardíaca um ano antes, durante uma cirurgia eletiva e com diagnóstico de STT, e que teve recuperada a função para o ventrículo esquerdo normal (VE), agora, 1 ano após o episódio, foi ao consultório de um dos autores solicitando uma avaliação de risco cardiovascular para outra cirurgia plástica eletiva.

Após cuidadosa seleção, foram analisados os dados dos seis estudos, nos quais foram considerados fatores com possível associação com a recorrência da STT. O sexo feminino era mais propenso a recidivas. A proximidade ao primeiro episódio da síndrome foi um fator descrito por alguns autores como predisponente a uma maior chance de recidiva. Baixo IMC e hipercontratilidade ventricular média também foram relatados como predisponentes à recorrência da STT. O uso de BB ou outros medicamentos para insuficiência cardíaca (IC) não foi comprovado na redução da chance de recorrência.

Alguns autores podem entender que novos episódios de STT não são recorrentes, mas sim um espectro clínico da doença. De acordo com consenso e declarações internacionais, o termo recorrência foi utilizado aqui pois, após o primeiro episódio, os pacientes recuperam sua função ventricular global e segmentar, recorrendo à disfunção no episódio subsequente.

Apenas um estudo desta revisão sistemática^10^ analisou o sexo como uma variável de recorrência, sem recorrência nos homens e uma taxa de recorrência nas mulheres de 14,8%. Em todos os outros estudos selecionados, como no restante da literatura,^6^ se não em todos, a maioria dos pacientes que apresentaram recidiva eram mulheres. Embora haja relatos de recorrência em homens,^14^ eles são extremamente raros. Esses dados sugerem fortemente que o sexo feminino é um fator predisponente à recorrência da STT.

Em relação à idade, Patel et al.^10^ encontraram maior taxa de recorrência entre mulheres com menos de 50 anos, quando comparadas às mulheres com 50 anos ou mais (16 *versus* 3%, respectivamente; p = 0,017). Isso sugere que as mulheres mais jovens tendem a ter recorrência da síndrome com mais frequência. No entanto, neste estudo, mulheres com menos de 50 anos apresentaram maior índice de distúrbios psiquiátricos, deixando em dúvida se o maior índice de recorrência da STT foi devido à menor idade ou à associação com distúrbios psiquiátricos. Vriz et al.^12^ relataram maior taxa de recorrência em pacientes idosos – pacientes com recidiva tinham idade média de 71,4 anos, enquanto aqueles com apenas um episódio tinham idade média de 65,7 anos. Uma revisão sistemática com meta-análise, composta por 31 estudos,6 encontrou uma idade média de 65,5 anos entre os pacientes que apresentaram recidiva, a maioria mulheres. Esses dados mostram a divergência entre diferentes estudos em relação à faixa etária que estaria mais predisposta a um episódio de recorrência da STT. Coortes com amostras maiores, abrangendo faixas etárias maiores, são necessárias para melhor esclarecer essa associação.

Quatro estudos desta revisão sistemática relataram o tempo entre o primeiro e o segundo episódio. Episódios de recorrência foram relatados por cerca de 22 dias^12^ até pouco mais de nove anos após o primeiro episódio.^3^ Embora exista um relato de recorrência até dez anos após o episódio inicial,^15^ casos como esse são extremamente raros. Looi et al.^9^ observaram que a recidiva foi mais frequente no primeiro ano após o episódio inicial. Elesber et al.^11^ apresentaram maior taxa de recorrência anual nos quatro primeiros anos em comparação aos anos subsequentes (2,9 *versus* 1,3% ao ano, respectivamente). Vriz et al.,^12^ relataram em seu estudo uma maior recorrência da síndrome nos primeiros três meses após o primeiro episódio. Outro estudo, publicado em 2017,^16^ relatou recorrência da STT em cinco pacientes, com o segundo episódio ocorrendo em média em 2,1 anos. Todos esses dados corroboram a ideia de que a probabilidade de recorrência da STT diminui com o tempo, sendo mais provável nos primeiros meses após o primeiro episódio, e há uma diminuição gradual nas chances de um segundo episódio ao longo dos anos, reduzindo significativamente após quatro anos.

Até o momento, o único estudo identificado nesta revisão que abordou uma associação entre recorrência da STT e IMC foi o de Nishida et al.^13^ Neste estudo, o baixo IMC foi um fator de risco para recorrência da STT. Quanto maior o IMC do indivíduo, menores as chances de recidiva, com uma HR de 0,75 (para cada aumento de 1kg/m^2^). Uma explicação clara para essa associação não foi possível, mas estudos recentes^17^^,^^18^ sugeriram que a resposta hemodinâmica ao estresse mental é mais intensa em pessoas com menor IMC, enquanto a atividade basal do sistema nervoso simpático desses indivíduos é menor do que nos indivíduos com maior IMC. Assim, pode-se sugerir que a maior sensibilidade do sistema nervoso simpático em pessoas com menor IMC reduziria seu limiar a um estresse emocional, desencadeando uma potencial STT.

Em relação à apresentação clínica dos pacientes que apresentaram STT, Looi et al.^9^ descreveram uma taxa absoluta mais alta de recidiva nos pacientes que apresentavam elevação do segmento ST no ECG quando comparados aos pacientes que não o apresentaram, com uma taxa de recorrência de 7,4 *versus* 6,3%, respectivamente; não houve significância estatística, p = 1,00. Outro estudo de Dib et al.^19^ relatou que não houve diferença na taxa de recorrência em 5 anos relacionada à apresentação do ECG, 13% naqueles que apresentaram elevação do segmento ST, 5% naqueles que apresentaram inversão da onda T e 17% naqueles com alterações inespecíficas no segmento ST e onda T (p = 0,25). Tais dados não sugerem que uma alteração eletrocardiográfica específica altere o prognóstico das pessoas afetadas pela STT em relação à sua recorrência.

O estudo de Nishida et al.^13^ mostrou inicialmente uma associação entre envolvimento biventricular e recorrência, mas nenhuma significância estatística foi alcançada nesta análise, p=0,06. Outro estudo, de Kagiyama et al.,^20^ também analisou essa relação com o padrão morfológico da síndrome manifestada pelos pacientes, sendo a taxa de recorrência em pacientes com envolvimento biventricular maior quando comparada àquelas com morfologia clássica, ou seja, 4,8 e 0%, respectivamente. Nishida et al.^13^ observaram maior taxa de recorrência em pacientes com obstrução ventricular média, o que corresponde à hipercontratilidade do terço médio do ventrículo esquerdo, ocorrida em pacientes com balão apical, provavelmente como mecanismo compensatório. Não foram encontrados mais estudos para avaliar essa relação.

Quatro estudos desta revisão sistemática analisaram o uso de BB como um possível método para prevenir a recorrência da STT. No estudo de Looi et al.,^9^ quatro (57%) dos pacientes estavam em uso de BB na recorrência; Templin et al.^3^ relataram que 29 pacientes (50,8%) estavam em uso de BB durante o segundo episódio; no estudo de Vriz et al.,^12^ a terapia com BB não impediu a recorrência; Elesber et al.^11^ mostraram uma taxa de recorrência de 80% entre os pacientes em uso de BB e 52% nos pacientes que não usavam esse tipo de medicamento, sem significância estatística (p = 0,10). Juntos, esses dados sugerem que a terapia com BB não está associada à prevenção de episódios de recorrência do STT. Elesber et al.^11^ também compararam a recorrência entre pacientes em e sem uso de aspirina, IECA/BRA e estatinas. Em seu estudo, os pacientes em uso de aspirina apresentaram uma taxa de recorrência de 60%, enquanto aqueles que não usaram este medicamento apresentaram uma taxa de recorrência de 67%, sem significância estatística (p = 0,67). A taxa de recorrência entre os pacientes que usaram e os que não usaram IECA/BRA foi de 60 e 51%, respectivamente (p = 0,59). Dos pacientes que apresentaram recidiva, 40% usaram estatinas e 33% não, nem houve significância estatística (p = 0,67). Diante do exposto, nenhum dos estudos desta revisão sistemática sugeriu terapia medicamentosa específica para prevenir a recorrência da STT. Em vários estudos, o uso de BB não mostrou eficácia na prevenção da STT. Esse medicamento também não se mostrou útil para esse fim em uma revisão sistemática com meta-análise de 31 estudos.^6^ No entanto, esse mesmo estudo6 mostrou associação negativa entre o uso de inibidores da ECA ou BRA e a taxa de recorrência, ou seja, o uso desses medicamentos diminuiu as taxas de recorrência, diferentes das encontradas por Elesber et al.^11^ Coortes segmentares de longo prazo com maior número de pacientes com STT e que fizeram uso de IECA/BRA são necessárias para esclarecer melhor essa associação.

O maior estudo desta revisão foi o de Templin et al.^3^ Este foi um estudo de caso-controle com 1.750 pacientes com STT de acordo com os critérios de Mayo e 57 casos de recorrência foram encontrados em longo prazo. Os autores tiveram como objetivo avaliar características clínicas, preditores prognósticos, evolução clínica e resultados da STT em uma ampla população. No entanto, como eles não se concentraram especificamente nos preditores de recorrência, o artigo não traz informações específicas sobre esse subgrupo, além do uso de BB.

Este artigo atualiza e complementa a revisão sistemática de Singh et al.^6^ Alguns dados podem ser confirmados nessa oportunidade, como a associação entre sexo feminino e maior taxa de recorrência e a não eficácia dos BB na prevenção de um segundo episódio. Outras variáveis, ainda não abordadas por Singh et al.,^5^ podem estar associadas a uma maior taxa de recorrência nessa revisão sistemática, como menor tempo após o primeiro episódio, baixo IMC e hipercontratilidade no terço médio do VE. Neste estudo, o STROBE^8^ foi utilizado para avaliar e selecionar os estudos encontrados, enquanto Singh et al. utilizaram o “*Quality of Reporting of Observational Longitudinal Research*”,^21^ e um foco especial foi dado à taxa de recorrência da STT, selecionando artigos que relatassem um mínimo de 3% da taxa de recorrência, o que pode ter transformado essa revisão sistemática em uma tarefa mais específica.

Dada a experiência local dos autores, foi observada prevalência da STT de 3,2% em pacientes inicialmente suspeitos de infarto agudo do miocárdio com supradesnivelamento do segmento ST e não houve recidivas na mediana do acompanhamento ambulatorial de 1 ano.

Não foram encontrados dados sobre o risco de recorrência quando submetidos ao mesmo fator de estresse. Se uma segunda exposição ao mesmo estressor deve ser evitada ou não pode ser um assunto de interesse para estudos futuros.

Entre as limitações desta revisão, como as dos estudos selecionados, estão: o fato de alguns estudos terem sido realizados em uma única população sem validação externa; a falta de mais detalhes clínicos dos pacientes que apresentaram recidiva (a maioria dos artigos não traz dados dos pacientes que se repetiram individualmente, portanto, não foi possível analisar os dados combinados dos pacientes individualmente); além da escassez de estudos relacionados ao tema, embora esse fator não tenha impedido a realização desta revisão sistemática. Uma metodologia sólida, com um alto ponto de corte no STROBE e o uso do modelo PRISMA forneceram uma base sólida para a construção consistente desta revisão sistemática.

## Conclusão

Considerando o exposto acima, sexo feminino, menor IMC, hipercontratilidade do terço médio do VE e menor tempo após o primeiro episódio foram associados a maior chance de recidiva. A idade e a apresentação eletrocardiográfica do paciente, relacionadas à manifestação de um segundo episódio da STT, merecem ser melhor investigadas por estudos com populações maiores.
