# Supplementary material for: Fatores Associados à Recorrência na Síndrome de Takotsubo: Uma Revisão Sistemática
Source: Arq Bras Cardiol. 2020 Apr 6;114(3):477–83. [Article in Portuguese] doi: 10.36660/abc.20180377 (PMC7792734; doi:10.36660/abc.20180377)
Supplement: Translation

# Translation: Factors Associated with Recurrence in Takotsubo Syndrome: A Systematic Review

**Authors:** Felipe Alverenga Duarte Campos, Luiz Eduardo Fonteles Ritt, João Paulo Soares Costa, Constança Margarida Cruz, Gilson Soares Feitosa-Filho, Queila Borges de Oliveira, Eduardo Sahade Darzé

**Affiliations:** 1 SalvadorBA Brazil Escola Bahiana de Medicina e Saúde Pública, Salvador, BA – Brazil; 2 SalvadorBA Brazil Hospital Cárdio Pulmonar, Salvador, BA – Brazil; 3 SalvadorBA Brazil Hospital Santo Antônio - Obras Sociais Irmã Dulce, Salvador, BA – Brazil; 4 SalvadorBA Brazil Faculdade de Tecnologia e Ciências, Curso de Medicina, Salvador, BA – Brazil

**Keywords:** Takotsubo Syndrome, Takotsubo cardiomyopathy, Recurrence

## Abstract

**Background:**

Takotsubo syndrome (TTS) is characterized by a temporary systolic dysfunction of the left ventricle (LV) related to a stressful event. However, the factors associated with its recurrence are still not well established.

**Objective:**

To analyze the main factors associated with TTS recurrence.

**Methods:**

A systematic review was performed using the PRISMA model. Observational studies, published between January 2008 and October 2017, which presented a recurrence rate of at least 3% and/or 5 or more patients with recurrence, and who met at least 80% of the STROBE criteria were included.

**Results:**

six articles reached the criteria to compose this systematic review. The recurrence rate ranged from 1 to 3.5% per year (global recurrence rate 3.8%). One study associated higher recurrence rate with the female gender, four reported the time between the first and second episodes, one study associated body mass index (BMI) and hypercontractility of the LV middle anterior wall to a higher recurrence rate. No association between recurrence and electrocardiographic changes were determined. Beta-blockers use was not associated with recurrence rates.

**Conclusions:**

Female gender, time from the first episode of the syndrome, low BMI and midventricular obstruction were reported as potential predictors of TTS recurrence.

## Preamble and case report

A 62-year-old female patient was admitted for elective rhytidoplasty and blepharoplasty surgeries. She weighed 61 kg and was 1.65 m tall, with a history of glaucoma and hypothyroidism. She was considered at low cardiovascular risk for the procedure, with no personal or family history of cardiovascular disease. During surgery, under general anesthesia, she presented idioventricular rhythm followed by circulatory shock and cardiorespiratory arrest. She was successfully resuscitated and her electrocardiography (ECG) showed an ST elevation pattern in lateral leads. She was promptly submitted to cardiac catheterization that showed no coronary lesion but akinesia in apical and medial ventricular walls and hyperkinesia of the basal parts, a pattern that resembles the Takotsubo Syndrome (TTS). This pattern was confirmed in an ECG which showed an ejection fraction of 40%. After adjustments for heart failure therapy, she was discharged from the hospital in 10 days, clinically stable. In six months, she had already recovered her global and segmental functions. After 1 year, she was planning to undergo another plastic surgery and asked about her recurrence risk. This question was the main drive for this systematic review.

## Introduction

TTS, also called Takotsubo cardiomyopathy or broken heart syndrome,^1^^,^^2^ is characterized by a temporary left ventricle systolic and diastolic dysfunction, usually associated with an event of great emotional or physical stress. It presents clinically with acute chest pain, dyspnea, electrocardiographic changes, and the presence of elevated cardiac injury biomarkers, being very similar to an acute coronary syndrome despite the absence of significant coronary stenosis related to the affected area.^3^ It is estimated that about 2% of patients with suspected acute coronary syndrome actually have TTS.^4^

Postmenopausal women are the group most affected by this condition, probably due to hormonal issues, although men and young people can also have TTS. It is suggested that the pathophysiology of the disease is related to a large and abrupt discharge of catecholamines.^2^ Therefore, the use of beta-blockers (BB) has been proposed as a prevention strategy.^3^

The prognosis is usually good and characterized as benign by many authors, even though there is a 1-2% risk of ventricular arrhythmias and approximately 2% of in-hospital mortality associated with TTS.^5^ Patients with a history of TTS have an annual recurrence rate of 1.5%, though it may be as high as 11% in 4 years.^3^^,^^5^^,^^6^

In recent years, there has been an increase in the number of published TTS-related studies, especially in the USA, Europe, and Japan. Much of the data on this pathology comes from the International Takotsubo Registry (InterTAK Registry), an international collaborative network with data from 35 cardiovascular centers in 15 different countries.^2^^,^^7^ However, predictors of TTS recurrence are still not well established.

## Objective

The present study aimed to analyze the main factors associated with TTS recurrence.

## Methods

A systematic review of the literature was proposed, using the PRISMA model. The main databases of international literature – PubMed, Scielo, Lilacs, and Cochrane – were searched.

As a search strategy, the following descriptors were used: Takotsubo Syndrome; Left Ventricular Apical Ballooning Syndrome; Takotsubo Cardiomyopathy; Stress Cardiomyopathy; Broken Heart Syndrome. The PubMed MeSH tool was used adding Recurrence as a complementary descriptor.

Strategy for articles selection: the selection was carried out in October 2017. All articles published between January 2008 and October 2017 were initially included for further appreciation. First, the titles were evaluated, followed by the abstract, and finally, a careful analysis of the complete article was conducted in order to identify its quality and relevance to the proposed objective. Strengthening the Reporting of Observational Studies in Epidemiology (STROBE) criteria^8^ for assessing the methodological quality of observational studies were used, with a minimum of 80% compliance of the 22 items in the STROBE checklist to be included in this study. This whole process was carried out by two researchers. Articles were also searched based on the references of the selected articles.

Only articles characterized as prospective cohort study, retrospective cohort study, case control or case series study were selected. Articles in which the text was not in English were excluded. Only studies reporting at least 3% recurrence rate and/or five or more patients with recurrence were included, so that there could be a significant analysis of the recurrence predictors.

## Results

Initially, a total of 164 articles were identified. Four other studies were identified and selected from the references of the initially identified articles. At the end of the analysis of the studies, six were selected to compose this systematic review (Figure 1 and Table 1).

**Figure 1:** Prisma Flowchart of the studies selection for the composition of the systematic review.

**Table 1:** Score and percentage of articles quality based on STROBE (Strengthening the Reporting of Observational Studies in Epidemiology) criteria

Reference	Study design	Publishing journal	Points on STROBE	%
Looi et al.,^9^	Prospective cohort.	Journal of Heart, Lung and Circulation.	18	81.8
Templin et al.,^3^	Case-control.	The New England Journal of Medicine.	19	86.3
Patel et al.,^10^	Retrospective cohort.	Journal of Cardiac Failure.	19	86.3
Elesber et al.,^11^	Retrospective cohort.	Journal of the American College of Cardiology.	19	86.3
Vriz et al.,^12^	Prospective cohort.	Journal of Cardiovascular Medicine.	18	81.8
Nishida et al.,^13^	Case-control.	Heart and Vessels.	20	90.9

Globally, the recurrence rate, before excluding the four articles that did not meet the criteria of at least 3% recurrence rate and/or five or more patients with recurrence, ranged from 0.2 to 5% per year. The global recurrence rate, considering the selected studies, was 3.8% in a follow up that ranged from 5 to 17 years.

Table 2 shows the main information for each selected article. Looi et al.^9^ studied a prospective cohort study of 100 patients diagnosed with TTS by the Mayo criteria. From these, seven patients (7%) had a recurrence and one presented with four recurrent episodes. Recurrences occurred between 99 and 679 days after the first episode. All recurrences occurred within two years after the first episode, being more frequent in the first year. In four of the seven patients who presented with recurrences (57%), the initial and subsequent events were triggered by emotional stress. Four of the seven patients who had recurrences were already using a BB in the second episode.

**Table 2:** Selected studies characteristics

Reference	Studied population	Level of Significance adopted	Recurrence rate (recurrence N/total N)	Analyzed data with possible recurrence association
Looi et al.,^9^	Patients admitted to Middlemore Hospital, Auckland City Hospital and North Shore Hospital, Auckland, New Zealand.	p < 0.05	7% (7/100)	– Time between manifestations: recurrences occurred from 99 to 679 days after the first episode, being more frequent in the first year. – Triggering factor: 57% of patients with recurrence presented emotional stress as a trigger. – Clinical characteristics: recurrence in patients presenting ST elevation was not higher when compared to patients who did not present ST elevation (7.4 and 6.3%, respectively); p = 1.00. – Medications in use: 4 (57%) of the patients used BB on recurrence.
Templin et al.,^3^	Patients obtained through the Mayo Clinic database. Patients with ACS from Zurich Acute Coronary Syndrome Registry.	p < 0.05	3.26% (57/1750)	– Time between manifestations: recurrence occurred from 25 days to 9.2 years after the first episode. – Medications in use: 29 patients (50.8%) used BB during the second episode.
Patel et al.,^10^	Patients obtained through the Mayo Clinic database.	p < 0.05 for men versus women p < 0.25 for comparison with women ≥ 50 years of age (due to multiple comparisons)	3.13% (7/224)	– Gender: there were no recurrences in men and all 7 recurrences were in women (14.8%) – Age: recurrence in women aged < 50 years was more prevalent in relation to recurrence in women aged ≥ 50 years (16 and 3%, respectively; p = 0.017).
Elesber et al.,^11^	Patients diagnosed with TTS submitted to the Mayo Clinic catheterization center database.	p < 0.05	11.4% (10/100)	– Time between manifestations: 4.4±4.6 mean years between episodes, with a higher recurrence rate in the first 4 years compared to subsequent years (2.9 and 1.3% a year, respectively). – Medications in use: recurrence in patients in use X without use of: aspirin (60x67%), p = 0.67; ACEI/ARB (60x51%), p = 0.59; BB (80x52%), p = 0.10; Statins (40x33%), p = 0.67.
Vriz et al.,^12^	Patients at San Antonio Community Hospital (San Daniele del Friuli, Udine, Italy).	p < 0.05	21.7% (5/23)	– Age: more frequent in older patients. – Time between manifestations: recurrence occurred on an average of 105.4 ± 82.92 days after the first episode, being more frequent in the first 3 months. – Clinical characteristics: more frequent recurrence in patients with lower LVEF, lower SBP and higher troponin peak. – Medications in use: therapy with BB did not prevent recurrence.
Nishida et al.,^13^	Patients from the BOREAS Registry database.	p < 0.05	2.8% (7/251)	-Clinical characteristics: low BMI, medium-ventricular hypercontractility and right ventricular involvement were both associated with a higher rate of recurrence of TTS (p = 0.048, 0.01, and 0.06, respectively). HRs of recurrence for BMI (per increase by 1 kg/cm^2^) and MVO were 0.75 (95% CI 0.54–0.99) and 14.71 (95% CI 1.87–304.66), respectively.

BB: beta-blocker; ACS: acute coronary syndrome; TTS: Takotsubo syndrome; ACEI: angiotensin converting enzyme inhibitor; ARB: angiotensin II receptor blocker; LVEF: left ventricle ejection fraction; SBP: systolic blood pressure; BMI: Body Mass Index; HRs: hazard ratios; CI: confidence interval; MVO: microvascular obstruction.

Templin et al.^3^ presented a case-control study with 1,750 TTS patients, according to the Mayo criteria. From these, 455 patients were matched, by age and gender, with patients diagnosed with acute coronary syndrome (ACS) and who had their data obtained through the Zurich Acute Coronary Syndrome Registry. During a 17-year follow-up period, 57 patients with TTS had recurrences, representing a rate of 1.8% recurrence per patient-year. The second episode occurred from 25 days to 9.2 years after the first one. A total of 29 of 57 patients with recurrences (50.8%) were on BB therapy at the time of their recurrent episode.

In the retrospective cohort study by Patel et al.,^10^ 224 patients diagnosed with TTS had their data obtained through the Mayo Clinic database over a 10-year period. Only 7 recurrent episodes were documented. None of the men had TTS recurrence. During a mean follow-up of 3.5 years, 2 women under 50 years of age (16%) and 5 women aged 50 years old or older (3%) developed TTS recurrence (p = 0.017).

Elesber et al.^11^ studied a retrospective cohort and analyzed data from 100 patients diagnosed with TTS over a period of 16 years and 11 months. Recurrence rate was 11.4% at a mean follow-up time of 4.4 ± 4.6 years, being higher in the first 4 years (2.9% per year), and decreasing to about 1.3% per year in subsequent segment time. There was no difference between patients with or without recurrences in relation to the use of: aspirin (60 *versus* 67%; p = 0.67); angiotensin converting enzyme inhibitor (ACEI)/angiotensin II receptor blocker (ARB) (60 *versus* 51%; p = 0.59); BB (80 *versus* 52%; p = 0.10); or statins (40 *versus* 33%; p = 0.67).

In another prospective cohort study,^12^ 23 patients who underwent coronary angiography were diagnosed with TTS according to the Mayo criteria, over a period of 7 years and 11 months. Five patients (21.7%) developed recurrent TTS and one patient presented with 2 recurrent episodes. The mean time to a recurrent episode was 105.4 ± 83 days, and the recurrence rate was higher in the first 3 months. Compared with patients with no recurrences, those with a recurrent episode were older (71.4 *versus* 65.7 years), had lower ejection fraction (36.5 *versus* 44.2%), higher systolic blood pressure (139 *versus* 128.4 mmHg) and higher peak troponin levels (8.1 *versus* 2.5 μg/ml). Three of the five patients who presented with a recurrence were on BB.

Nishida et al.^13^ presented a case-control study. Data from 251 patients who composed the BOREAS Registry (with 15 participating countries) from June 1999 to March 2012, were analyzed. Patients were divided into two groups, those with apical ballooning (type A), classic TTS presentation, and those with non-apical ballooning (non-A type), which included all other presentation forms of the syndrome. During a follow-up of 2.6 ± 2.8 years, the recurrence rate was 2.8% (7/251), with no significant difference between A and non-A groups (2.8 and 2.9 %, respectively). In the univariate analysis, low Body Mass Index (BMI) (p = 0.048), midventricular (p = 0.01), and concomitant right ventricular involvement (p = 0.06) were associated with TTS recurrence. Only BMI (hazard ratio [HR] 0.75; 95% confidence interval [CI] 0.54–0.99; p = 0.048) and midventricular obstruction (HR14.71; 95% CI 1.87–304.66; p = 0.01) remained significantly associated with TTS recurrence.

## Discussion

TTS recurrence rate is variable in the literature and the factors associated with it were not clearly defined as well. This statement was clear when, recently, that 62-year-old female patient who had a history of being resuscitated for cardiac arrest a year before during an elective surgery and had a diagnosis of TTS, and who recovered to normal left ventricular (LV) function, now 1 year after the index episode, came to the office of one of the authors asking for a cardiovascular risk evaluation for another elective plastic surgery.

After careful selection, data from the six studies were analyzed, in which factors with possible association with TTS recurrence were considered. The female gender was more prone to recurrence. Proximity to the first episode of the syndrome was a factor described by some authors as predisposing to a greater chance of recurrence. Low BMI and medium-ventricular hypercontractility were also reported as predisposing to TTS recurrence. Use of BB or other heart failure (HF) medications was not proven to reduce the chance of recurrence.

Some authors may understand that new episodes of TTS are not recurrence, but instead a clinical spectrum of the disease. In accordance with international consensus and statements, the term recurrence was used here as after the first episode patients recover their global and segmental ventricular function recurring the disfunction in the subsequent episode.

Only one study of this systematic review^10^ analyzed gender as a recurrence variable, with no recurrence in men and a recurrence rate in women of 14.8%. In all other studies selected, as in the rest of the literature,^6^ if not all, most patients who had recurrence were women. Although there are reports of recurrence in men,^14^ they are extremely rare. These data strongly suggest that female gender is a predisposing factor to TTS recurrence.

In relation to age, Patel et al.^10^ found a higher rate of recurrence among women aged less than 50 years, when compared to women aged 50 years old or older (16 *versus* 3%, respectively; p = 0.017). This suggests that younger women tend to have recurrence of the syndrome more often. However, in this study, women younger than 50 years of age had higher rate of psychiatric disorders, leaving in doubt whether the higher rate of TTS recurrence was due to lower age or to association with psychiatric disorders. Vriz et al.^12^ reported higher recurrence rate in older patients – patients who had recurrence had a mean age of 71.4 years, while those with only one episode had a mean age of 65.7 years. A systematic review with meta-analysis, composed of 31 studies,^6^ found an average age of 65.5 years among patients who presented recurrence, most of them women. These data show the divergence between different studies in relation to the age group that would be more predisposed to an episode of TTS recurrence. Cohorts with greater samples, covering larger age groups, are necessary to better clarify this association.

Four studies of this systematic review reported the time between the first and second episodes. Episodes of recurrence have been reported for about 22 days^12^ until slightly more than nine years after the first episode.^3^ Although there is a recurrence report up to ten years after the initial episode,^15^ cases like this are extremely rare. Looi et al.^9^ found that recurrence was more frequent in the first year after the initial episode. Elesber et al.^11^ showed higher annual recurrence rate in the first four years compared to subsequent years (2.9 *versus* 1.3% per year, respectively). Vriz et al.^12^ reported in their study a higher recurrence of the syndrome in the first three months after the first episode. Another study, published in 2017,^16^ reported TTS recurrence in five patients, with the second episode occurring in an average of 2.1 years. All these data corroborate the idea that a person's likelihood of TTS recurrence decreases over time, being more likely in the first few months following the first episode, and there is a gradual decrease in the chances of a second episode over the years, reducing significantly after four years.

To date, the only study identified in this review that conducted an association between TTS recurrence and BMI was the one by Nishida et al.^13^ In this study, low BMI was a risk factor for TTS recurrence. The higher the BMI of the individual the lower his chances of recurrence, with a HR of 0.75 (for each 1kg/m^2^ increase). A clear explanation for this association was not possible, but recent studies^17^^,^^18^ have suggested that the hemodynamic response to mental stress is more intense in people with lower BMI, while the basal activity of the sympathetic nervous system of these individuals is lower than in individuals with higher BMI. Thus, one may suggest that the greater sensitivity of the sympathetic nervous system in people with lower BMI would reduce their threshold to emotional stress, triggering potential TTS.

Regarding the clinical presentation of the patients who presented TTS, Looi et al.^9^ described an absolute higher rate of recurrence in patients presenting ST elevation in their ECG when compared to patients who did not present it, with a recurrence rate of 7.4 *versus* 6.3%, respectively; there was no statistical significance, p = 1.00. Another study by Dib et al.^19^ reported that there was no difference in the 5-year recurrence rate related to ECG presentation, 13% in those who presented ST segment elevation, 5% in those who presented with T wave inversion, and 17% in those whit non-specific changes in the ST segment and T wave (p = 0.25). Such data do not suggest that a specific electrocardiographic alteration changes the prognosis of those affected by TTS with respect to its recurrence.

The study by Nishida et al.^13^ initially showed an association between biventricular involvement and recurrence, but no statistical significance was reached in this analysis, p = 0.06. Another study, by Kagiyama et al.,^20^ also analyzed this relationship with the morphological pattern of the syndrome manifested by the patients, being the recurrence rate in patients with biventricular involvement greater when compared to those with classic morphology, that is, 4.8 and 0%, respectively. Nishida et al.^13^ found higher recurrence rate in patients with medium ventricular obstruction, which corresponds to hypercontractility of the middle third of the left ventricle, which occurred in patients with apical ballooning, probably as a compensatory mechanism. No further studies were found to evaluate this relationship.

Four studies of this systematic review analyzed the use of BB as a possible method of preventing TTS recurrence. In the Looi et al.^9^ study, four (57%) of the patients were in use of BB on recurrence; Templin et al.^3^ reported that 29 patients (50.8%) were in use of BB during the second episode; in the study by Vriz et al.,^12^ BB therapy did not prevent recurrence; Elesber et al.^11^ showed a recurrence rate of 80% among patients on BB and 52% in patients who did not use this medication, without statistical significance (p = 0.10). Together, these data suggest that BB therapy is not associated to prevention of episodes of TTS recurrence. Elesber et al.^11^ also compared recurrence among patients in and without use of aspirin, ACEI/ARB, and statins. In their study, patients taking aspirin had a recurrence rate of 60%, whereas those who did not use this medication had a 67% recurrence rate, with no statistical significance (p = 0.67). The recurrence rate between patients who did and those who did not use ACEI/ARB was 60 and 51%, respectively (p = 0.59). From the patients who presented recurrence, 40% used statins and 33% did not, nor was there any statistical significance (p = 0.67). Given the above, none of the studies of this systematic review suggested specific drug therapy to prevent TTS recurrence. In several studies, the use of BB showed no efficacy in TTS prevention. This medication was also not useful for this purpose in a systematic review with a meta-analysis of 31 studies^6^. However, this same study^6^ showed a negative association between the use of ACE inhibitors or ARB and the recurrence rate, that is, the use of these medications decreased recurrence rates, different from those found by Elesber et al.^11^ Long-term segmental cohorts with a greater number of patients who presented TTS and made use of ACEI/ARB are necessary to better clarify this association.

The largest study in this review was the one by Templin et al.^3^ This was a case-control study with 1,750 TTS patients according to the Mayo criteria and 57 recurrences cases were found in the long term follow up. The authors aimed to evaluate clinical features, prognostic predictors, clinical course, and outcomes of TTS in a wide population. However, as they did not focus specifically on recurrence predictors, the article does not bring specific insights about this subgroup apart from the use of BB.

This article updates and complements the systematic review by Singh et al.^6^ Some data could be confirmed in this opportunity, such as the association between female gender and higher recurrence rate, and the non-efficacy of BB in the prevention of a second episode. Other variables, not yet addressed by Singh et al.,^5^ could be associated with a higher rate of recurrence by this systematic review, such as the shorter time after the first episode, low BMI and LV middle third hypercontractility. In this study, the STROBE^8^ was used in order to evaluate and select the studies found, while Singh et al. used the “Quality of Reporting of Observational Longitudinal Research”,^21^ and special focus was given to TTS recurrence rate, selecting articles that reported a minimum of 3% of recurrence rate, which may have turned this systematic review into more task-specific.

Given the local experience of the authors, a TTS prevalence of 3.2% was observed in patients initially suspected of ST elevation acute myocardial infarction and there were no recurrences in the 1-year ambulatory median follow up.

No data about the risk of recurrence when submitted to the same stress factor again were found. Whether a second exposure to the same stressor should be avoided may be a matter of interest for future studies.

Among the limitations of this review, as those of the selected studies, are: the fact that some studies were performed in a single population without external validation; the lack of more clinical details of the patients who presented recurrence (most articles do not bring data from the patients that recurred in an individual basis, thus it was not possible to analyze the combining data from individual patients together); in addition to the scarcity of studies related to the topic, although this factor did not prevent this systematic review realization to be carried out. A strong methodology, with a high cut-off point in STROBE and the use of the PRISMA model, provided a solid basis for consistently constructing this systematic review.

## Conclusion

Considering the above, female gender, lower BMI, LV middle third hypercontractility, and shorter time after the first episode were associated to a greater recurrence chance. Patient's age and electrocardiographic presentation, related to the manifestation of a second TTS episode, deserve to be better investigated by studies with larger populations.
